# Efficacy and safety of posaconazole for the prevention of invasive fungal infections in immunocompromised patients: a systematic review with meta-analysis and trial sequential analysis

**DOI:** 10.1038/s41598-020-71571-0

**Published:** 2020-09-03

**Authors:** Tse Yee Wong, Yee Shen Loo, Sajesh Kalkandi Veettil, Pei Se Wong, Gopinath Divya, Siew Mooi Ching, Rohit Kunnath Menon

**Affiliations:** 1 School of Pharmacy, International Medical University, Kuala Lumpur, Malaysia; 2 Department of Pharmacy Practice, School of Pharmacy, International Medical University, Kuala Lumpur, Malaysia; 3 Oral Diagnostic and Surgical Sciences, School of Dentistry, International Medical University, Kuala Lumpur, Malaysia; 4 Department of Family Medicine, Faculty of Medicine and Health Sciences, Universiti Putra Malaysia, Serdang, Malaysia; 5 Division of Clinical Dentistry, School of Dentistry, International Medical University, Kuala Lumpur, Malaysia

**Keywords:** Health care, Medical research

## Abstract

Invasive fungal infections are a potentially life-threatening complication in immunocompromised patients. The aim of this study was to assess the efficacy and safety of posaconazole as compared with other antifungal agents for preventing invasive fungal infections in immunocompromised patients. Embase, CENTRAL, and MEDLINE were searched for randomized conweekmonthtrolled trials (RCTs) up to June 2020. A systematic review with meta-analysis of RCTs was performed using random-effects model. Trial sequential analysis (TSA) was conducted for the primary outcome to assess random errors. A total of five RCTs with 1,617 participants were included. Posaconazole prophylaxis was associated with a significantly lower risk of IFIs (RR, 0.43 [95% CI 0.28 to 0.66, p = 0.0001]) as compared to other antifungal agents. No heterogeneity was identified between studies (I^2^ = 0%). No significant associations were observed for the secondary outcomes measured, including risk reduction of invasive aspergillosis and candidiasis, clinical failure, all-cause mortality, and treatment-related adverse events, except for infection-related mortality (RR, 0.31 [95% CI 0.15 to 0.64, p = 0.0001]). Subgroup analysis favoured posaconazole over fluconazole for the prevention of IFIs (RR, 0.44 [95% CI 0.28 to 0.70, p = 0.0004]). TSA confirmed the prophylactic benefit of posaconazole against IFIs. Posaconazole is effective in preventing IFIs among immunocompromised patients, particularly those with hematologic malignancies and recipients of allogenic hematopoietic stem cell transplantation.

## Introduction

Invasive fungal infections (IFIs) remain a significant health threat in immunocompromised individuals, including blood cancer patients and transplant recipients^1,2^. In addition to prolonged hospital stays and increased healthcare costs, high mortality rates are reported in affected patients^3^. In view of the substantial disease burden associated with IFIs, primary antifungal prophylaxis is crucial in patients at high risk of prolonged neutropenia.

Triazoles are an important class of antifungals in clinical settings due to their effectiveness and availability for oral administration^4,5^. Structurally related to itraconazole, posaconazole is an extended-spectrum second-generation triazole with improved potency^6^. The azoles inhibit the cytochrome P450 lanosterol 14α-demethylase (CYP51) enzyme thereby blocking the synthesis of ergosterol and disrupting fungal cell membrane integrity^4–6^. The long side chain of posaconazole enables enhanced hydrophobic binding to CYP51, resulting in activity against many fluconazole- and voriconazole-resistant isolates^6,7^. Posaconazole demonstrates excellent antifungal activity against *Candida* and *Aspergillus* species^7,8^, which are known to be the predominant fungal pathogens of IFIs^9,10^. In contrast to older triazoles, posaconazole offers an additional coverage against Mucorales^8,11^.

To date, randomized controlled trials (RCTs) investigating the role of posaconazole in primary prophylaxis have demonstrated variable results^12–16^. However, current evidence on the efficacy and safety of posaconazole as compared to other antifungal agents has not been comprehensively evaluated. The objective of the current systematic review with meta-analysis and trial sequential analysis (TSA) was to provide reliable estimates on the efficacy and safety of posaconazole from RCTs to facilitate evidence-based decision-making on its prophylactic use in immunocompromised patients.

## Methods

### Study design

The protocol of this systematic review was registered in the International Prospective Register of Systematic Reviews (PROSPERO) under CRD42019148129. This study was performed based on the Cochrane Handbook for Systematic Reviews of Interventions^17^ and was reported in accordance with the Preferred Reporting Items for Systematic Reviews and Meta-Analyses (PRISMA)^18^.

### Data sources

A systematic search for RCTs was performed in Embase, Cochrane Central Register of Controlled Trials (CENTRAL), and MEDLINE from inception to June 2020. Our search strategy included terms such as “immunocompromised host”, “leukemia”, “lymphoma”, “myelodysplastic syndromes”, “chemotherapy”, “transplants”, “graft vs host disease”, “invasive fungal infections”, “aspergillosis”, “candidiasis”, and “posaconazole”. The search was limited to human studies. A detailed description of the search strategy is provided in Supplementary material, Table 1.

### Study selection

Studies included were RCTs that met the following eligibility criteria: study participants of any age who were at risk of prolonged neutropenia (patients with hematologic malignancies who received chemotherapy or transplant recipients under immunosuppressive treatment); intervention was posaconazole at any dose; comparators were any other antifungal agents; primary outcome was the incidence of proven/probable IFIs, categorized as per the revised criteria by the European Organization for the Research and Treatment of Cancer and the Mycoses Study Group (EORTC/MSG)^19^; secondary outcomes were the incidence of invasive aspergillosis, incidence of invasive candidiasis, clinical failure, all-cause mortality, infection-related mortality, and treatment-related adverse events.

### Data extraction and quality assessment

Data extraction was performed independently by two reviewers (TYW and YSL) and transferred into a standardized data collection form. The extracted data included the study design, characteristics of the study participants, interventions, outcome definitions, and outcome measures. Data for all outcomes were extracted following the intention-to-treat principle. The risk of bias for every study was assessed independently by two reviewers (TYW and YSL) using the revised Cochrane Risk of Bias tool (RoB 2.0)^20^. Any disagreements were resolved by consensus among the reviewers.

### Data synthesis and statistical analysis

In the primary meta-analysis, we compared posaconazole with other antifungal agents in terms of efficacy in reducing the risk of proven/probable IFIs. Random-effects model was used to generate pooled relative risk (RR) and the corresponding 95% confidence interval (95% CI). Statistical significance was considered at a two-tailed p-value < 0.05. Comparison was made between posaconazole and fluconazole in the subgroup analysis. Multiple sensitivity analyses were carried out to assess the robustness of results from our primary meta-analysis, by employing fixed-effects model and excluding trials with high risk of bias. Heterogeneity between the trials was quantitatively assessed using I^2^ statistic, whereby I^2^ estimate ≥ 50% was indicative of substantial heterogeneity^17^. Funnel plot asymmetry testing and Egger’s regression test were performed to assess publication bias^21^. A p-value of < 0.05 for Egger’s test was considered as statistical evidence of significant small-study effects. All statistical analyses were performed using STATA software version 15.0 (StataCorp, College Station, TX, USA).

Meta-analyses that include a small number of studies or study participants, may produce false positive results (type I error) due to random errors^22^. TSA adjusts the threshold for statistical significance by considering the accrued sample size from all included trials in the cumulative meta-analysis and provides the required information size to determine the reliability of conclusions obtained from a meta-analysis^22,23^. Therefore, we conducted a TSA to assess the effect of random errors on our primary meta-analysis using an exclusive software developed by the Copenhagen Trial Unit (available at https://www.ctu.dk)^24^. Control event rate and anticipated relative risk reduction from our primary meta-analysis were used to perform TSA. For zero-event trials, constant continuity correction method was applied by adding a correction factor of 0.5 to the number of events and patients in both the treatment and control arms^25^.

The quality of evidence derived from meta-analytic estimates was rated (high, moderate, low or very low) based on the Grading of Recommendations Assessment, Development and Evaluation (GRADE) approach by using GRADEpro GDT software online (available at https://gradepro.org). The certainty of effect estimates was evaluated by considering the study design, risk of bias, inconsistency, indirectness, imprecision, and publication bias^26,27^.

### Ethics approval and consent to participate

Not applicable.

### Consent for publication

Not applicable.

## Results

### Included studies and main characteristics

**Figure 1:**
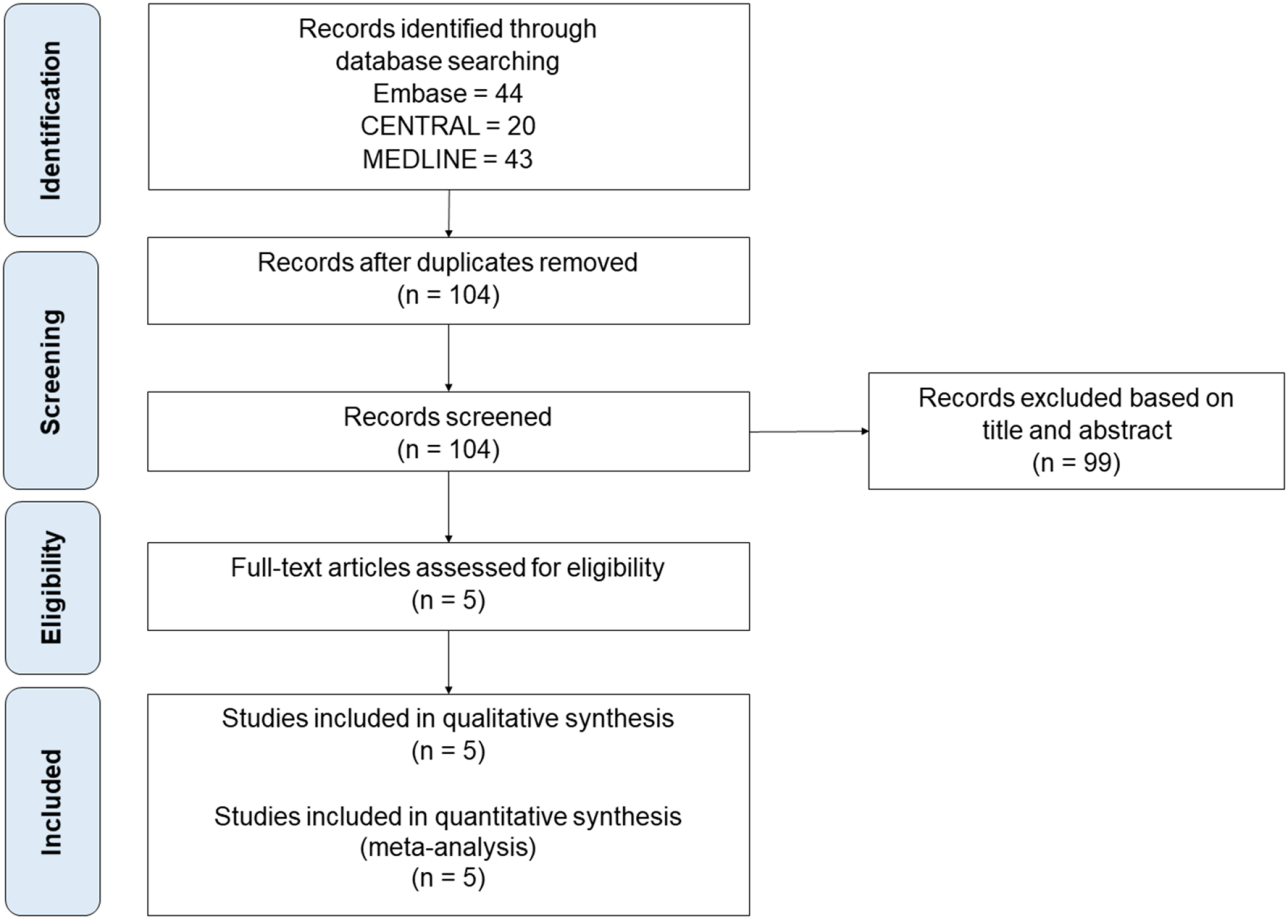
PRISMA flowchart for systematic literature search and study selection.

The process of identification, screening, and selection of studies is depicted in Fig. 1. The initial literature search identified 100 studies. After the removal of duplicates, 98 studies were screened for their eligibility. Of these, five RCTs^12–16^ met the eligibility criteria and were included in our primary meta-analysis. Table 1 describes the study characteristics of the included trials. A total of 1,617 participants aged ≥ 13-year-old with hematologic malignancies were included in this study. Three RCTs^12,15,16^ recruited patients who received chemotherapy and two RCTs^13,14^ enrolled patients who had undergone hematopoietic stem cell transplantation (HSCT) with/without the development of graft-versus-host disease (GVHD). Posaconazole oral suspension was used at a dose of 600 mg/day in most trials^12–15^, except one trial^16^ using 800 mg/day.

**Table 1:** Characteristics of included studies.

Year	Author	Trial design	Population	Age of study participants, median; range (year)	Interventions		Total duration of follow-up (week)	Endpoints^a^
					Treatment arm (n)	Control arm (n)		
2007	Cornely et al.^12^	Multicenter, single-blind, parallel-group	Patients with AML or MDS treated with chemotherapy	Treatment arm: 53; 13–82 Control arm: 53; 13–81	Posaconazole oral suspension 200 mg TDS (n = 304)	Fluconazole oral suspension 400 mg OD (n = 240) OR Itraconazole oral solution 200 mg BD (n = 58)	16	1–7
2007	Ullmann et al.^13^	Multicenter, double-blind, double-dummy, parallel-group	Patients with hematologic malignancies who had undergone allogenic HSCT and developed GVHD	Treatment arm: 42.2^b^; 13–72 Control arm: 40.4^b^; 13–70	Posaconazole oral suspension 200 mg TDS + placebo capsule OD (n = 301)	Fluconazole capsule 400 mg OD + placebo oral suspension TDS (n = 299)	24	1, 2, 3, 5, 6, 7
2012	Chaftari et al.^14^	Single-center, open-label, parallel-group	Patients with hematologic malignancies who had undergone allogenic HSCT	Treatment arm: 55; 20–66 Control arm: 56; 21–69	Posaconazole oral suspension 200 mg TDS (n = 24)	Amphotericin B lipid complex 7.5 mg/kg once per week (n = 22)	8	1, 4, 7
2013	Shen et al.^15^	Multicenter, open-label, parallel-group	Patients with AML or MDS treated with chemotherapy	Treatment arm: 40; 17–61 Control arm: 40; 15–68	Posaconazole oral suspension 200 mg TDS (n = 129)	Fluconazole 400 mg OD (n = 123)	16	1, 4, 5
2018	Epstein et al.^16^	Single-center, open-label, parallel-group	Patients with hematologic malignancies treated with chemotherapy	Treatment arm: 59; 26–74 Control arm: 61; 32–75	Posaconazole oral suspension 400 mg BD (n = 58)	IV Micafungin 100 mg OD (n = 59)	12	1–6

AML, acute myeloid leukemia; BD, twice daily; GVHD, graft-versus-host disease; HSCT, hematopoietic stem cell transplantation; IV, intravenous; MDS, myelodysplastic syndrome; n, number of randomized study participants; OD, once daily; TDS, three times daily.

^a^Study endpoints are listed as follow: 1—incidence of proven/probable invasive fungal infections; 2—incidence of invasive aspergillosis; 3—incidence of invasive candidiasis; 4—clinical failure; 5—all-cause mortality; 6—infection-related mortality; 7—treatment-related adverse events.

^b^Mean age.

### Risk of bias assessment

The risk of bias of all included trials is presented in Supplementary material, Fig. 1. Overall, one trial^14^ had low risk of bias, three trials^12,13,16^ had some concerns of bias, and one trial^15^ had high risk of bias. Majority of the trials^12,13,15,16^ did not provide information on randomization methods and allocation concealment. Although three trials^14–16^ followed an open-label design, outcome measurement was unlikely to be biased since the study endpoints were mostly binary outcomes that involved diagnostic procedures.

### Primary efficacy outcome: incidence of proven/probable IFIs

**Figure 2:**
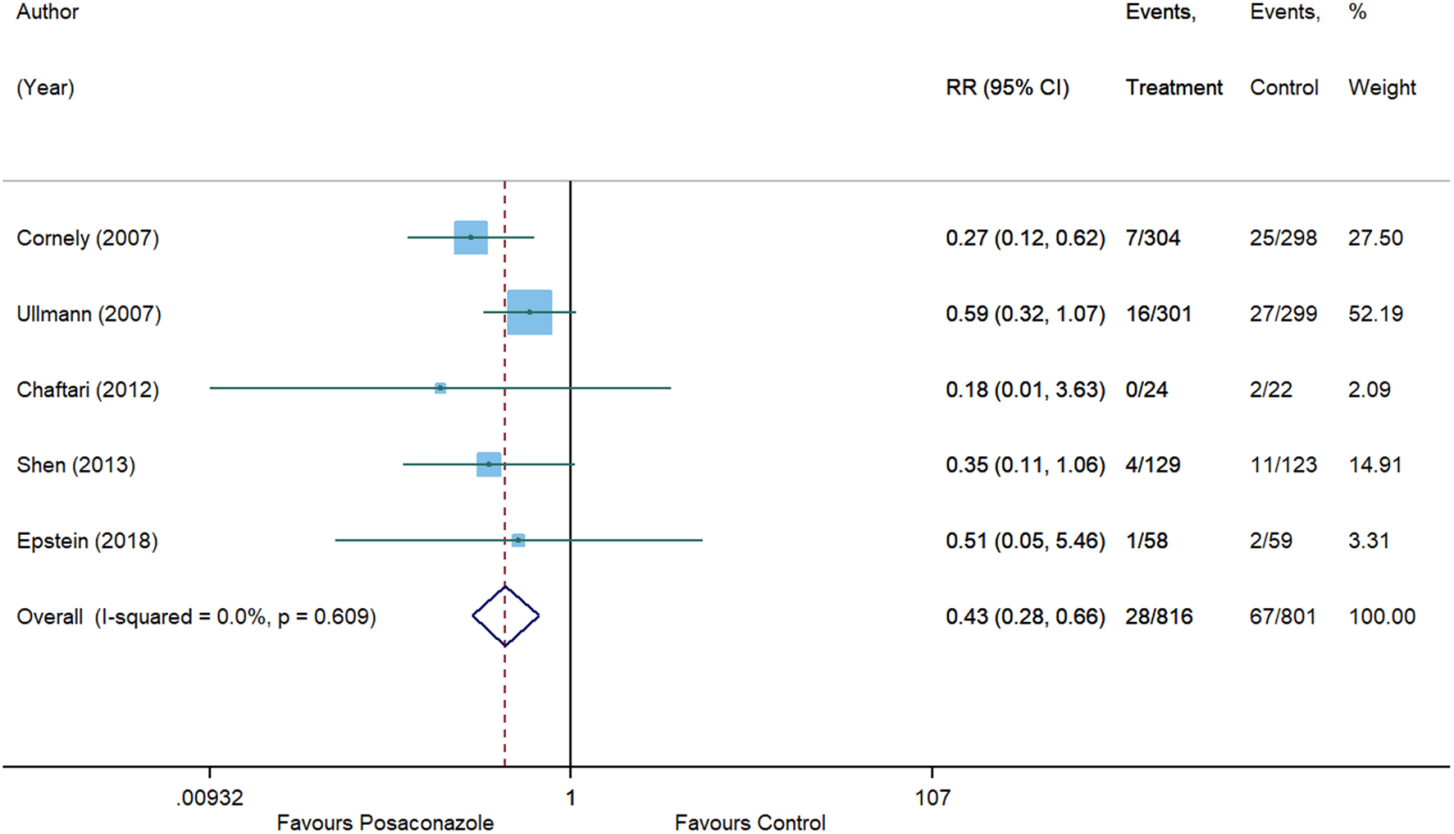
Forest plot and pooled risk estimate for the incidence of invasive fungal infections comparing posaconazole with other antifungal agents. RR, relative risk; 95% CI, 95% confidence interval.

**Figure 3:**
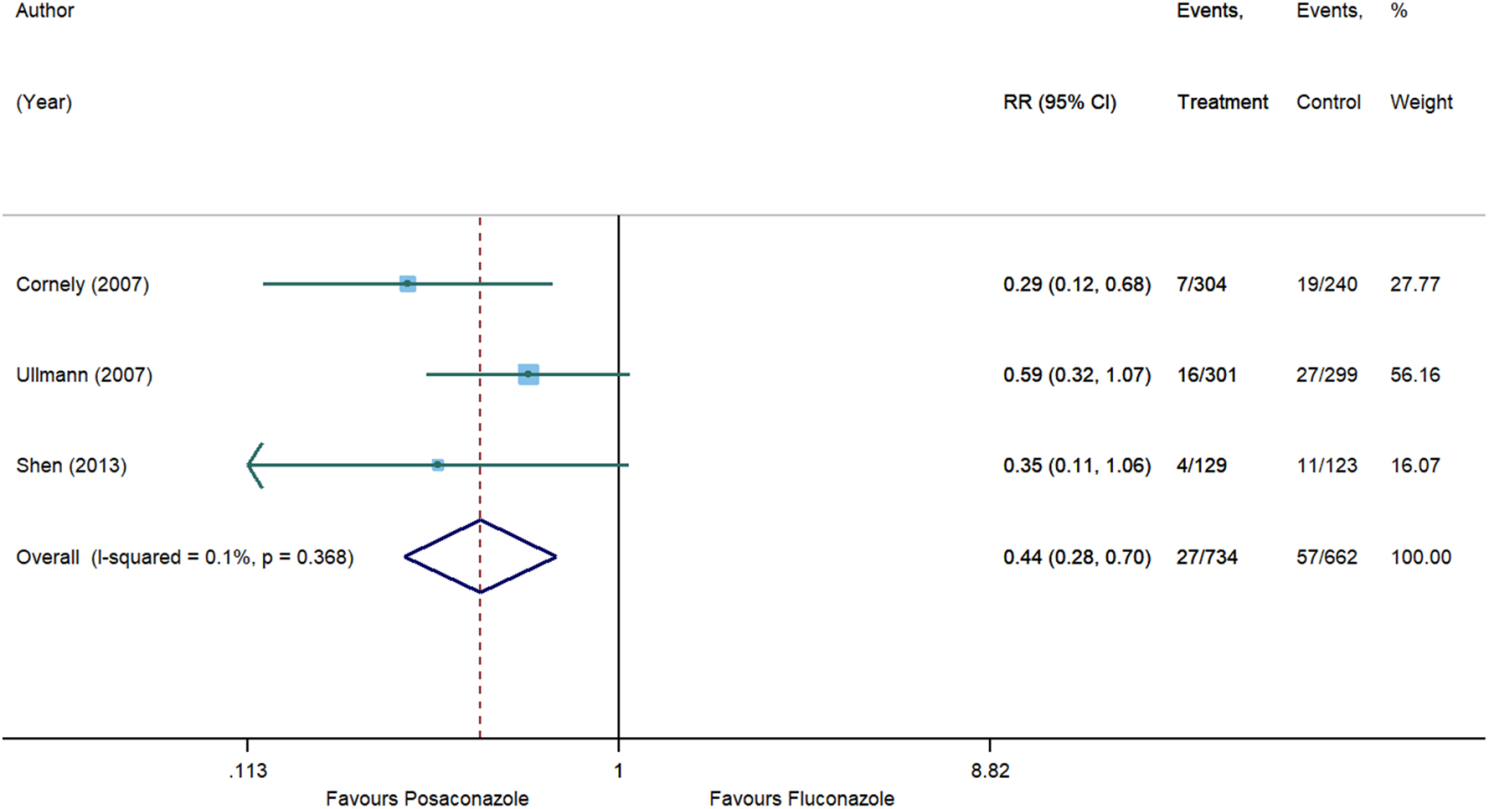
Forest plot and pooled risk estimate for the incidence of invasive fungal infections comparing posaconazole with fluconazole. RR, relative risk; 95% CI, 95% confidence interval.

Posaconazole demonstrated a statistically significant 57% reduction in risk of IFIs as compared to other antifungal agents (RR, 0.43 [95% CI 0.28 to 0.66], p = 0.0001, I^2^ = 0%) with no heterogeneity between studies (Fig. 2). Results from the subgroup analysis comparing posaconazole and fluconazole are depicted in Fig. 3.

### Sensitivity analyses

Findings from sensitivity analyses were consistent with our primary meta-analysis. Posaconazole was significantly more effective than other antifungal agents in preventing IFIs when a fixed-effects model was applied (RR, 0.41 [95% CI 0.27 to 0.63], p = 0.00005, I^2^ = 0%) (Supplementary material, Fig. 2) and after excluding one trial^15^ with high risk of bias (RR, 0.44 [95% CI 0.28 to 0.71], p = 0.0007, I^2^ = 0%) (Supplementary material, Fig. 3). Per-protocol analysis was not feasible as some trials^14,15^ did not report the number of participants who had completed the treatment phase.

### Trial sequential analysis

**Figure 4:**
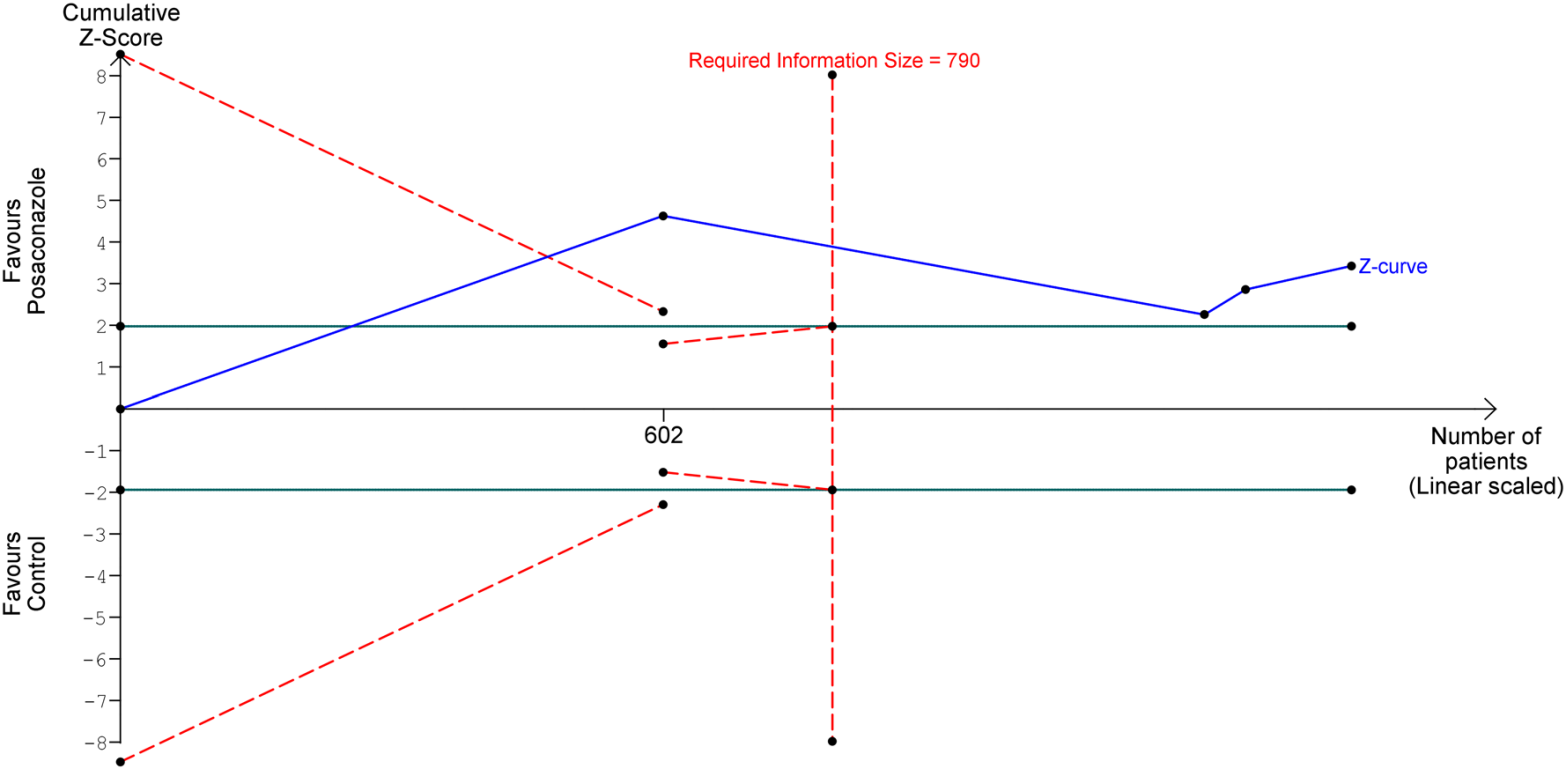
Trial sequential analysis evaluating the effect of posaconazole prophylaxis on the incidence of invasive fungal infections using random-effects meta-analysis.

By using a median event proportion in the control group of 8.71% after excluding one trial^15^ with high risk of bias, an alpha of 5% (two-sided), and a power of 80%, the required information size to demonstrate or reject a 56% relative risk reduction of IFIs with posaconazole prophylaxis was 790 study participants (Fig. 4). The number of participants included in the primary meta-analysis surpassed the required information size. In addition, the cumulative Z-curve crossed both the conventional and trial sequential monitoring boundaries, suggesting that the evidence was significant and conclusive.

### Publication bias

Funnel plot (Supplementary material, Fig. 4) showed weak asymmetry and Egger’s regression test (p = 0.442) (Supplementary material, Table 2) indicated no evident publication bias^28^, although the number of studies included in the primary meta-analysis was small.

### Secondary efficacy outcomes

**Figure 5:**
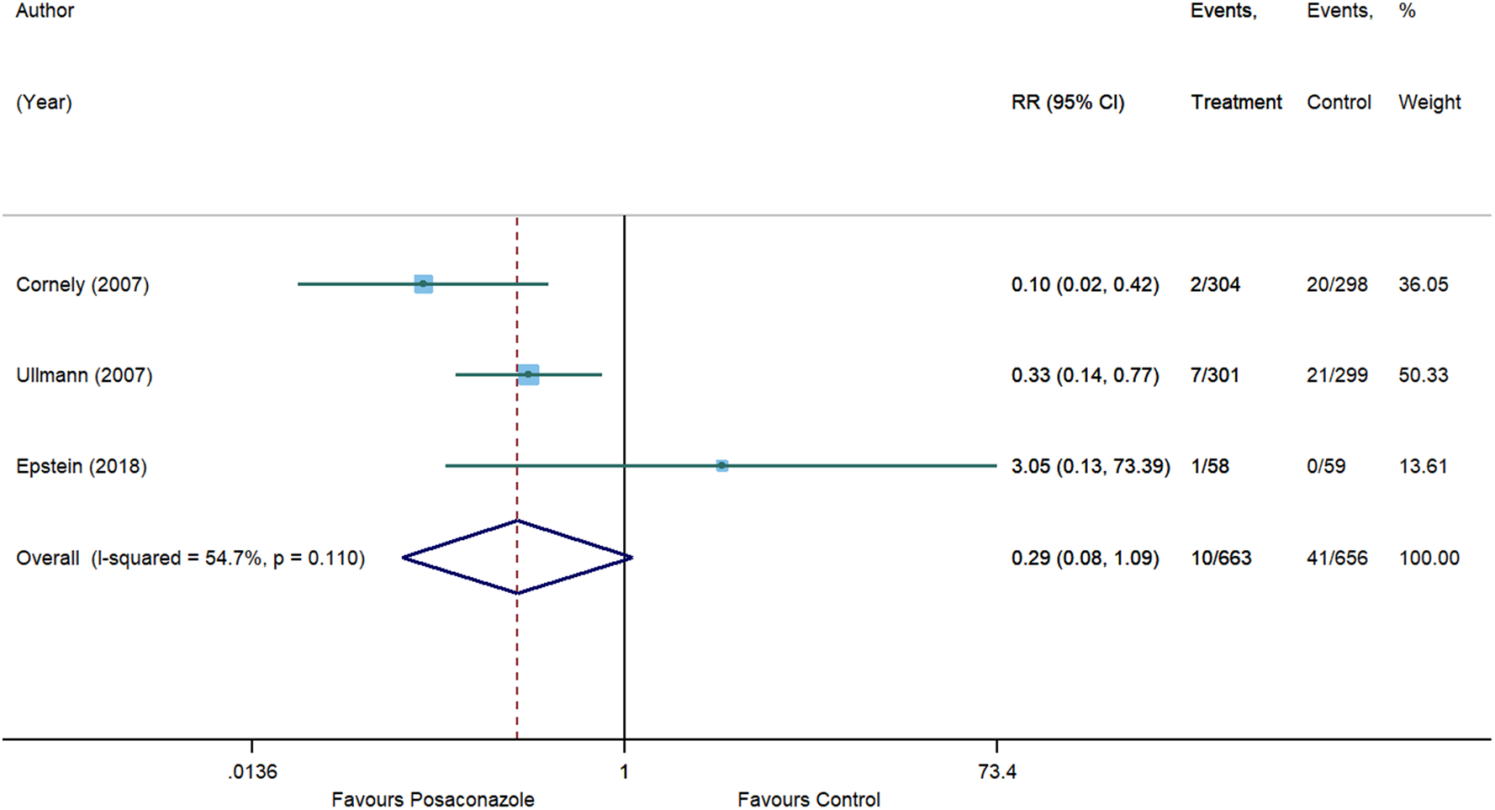
Forest plot and pooled risk estimate for the incidence of invasive aspergillosis comparing posaconazole with other antifungal agents. RR, relative risk; 95% CI, 95% confidence interval.

**Figure 6:**
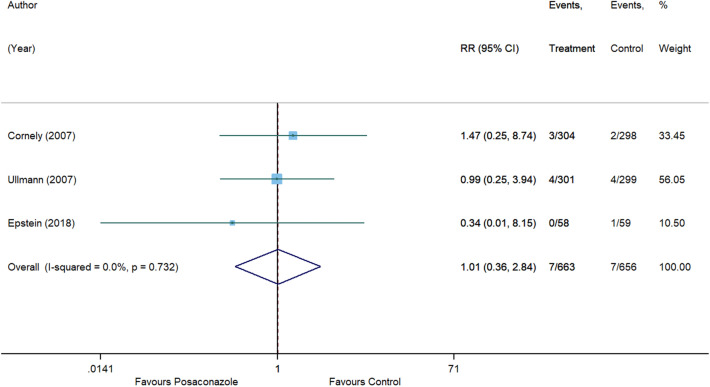
Forest plot and pooled risk estimate for the incidence of invasive candidiasis comparing posaconazole with other antifungal agents. RR, relative risk; 95% CI, 95% confidence interval.

**Figure 7:**
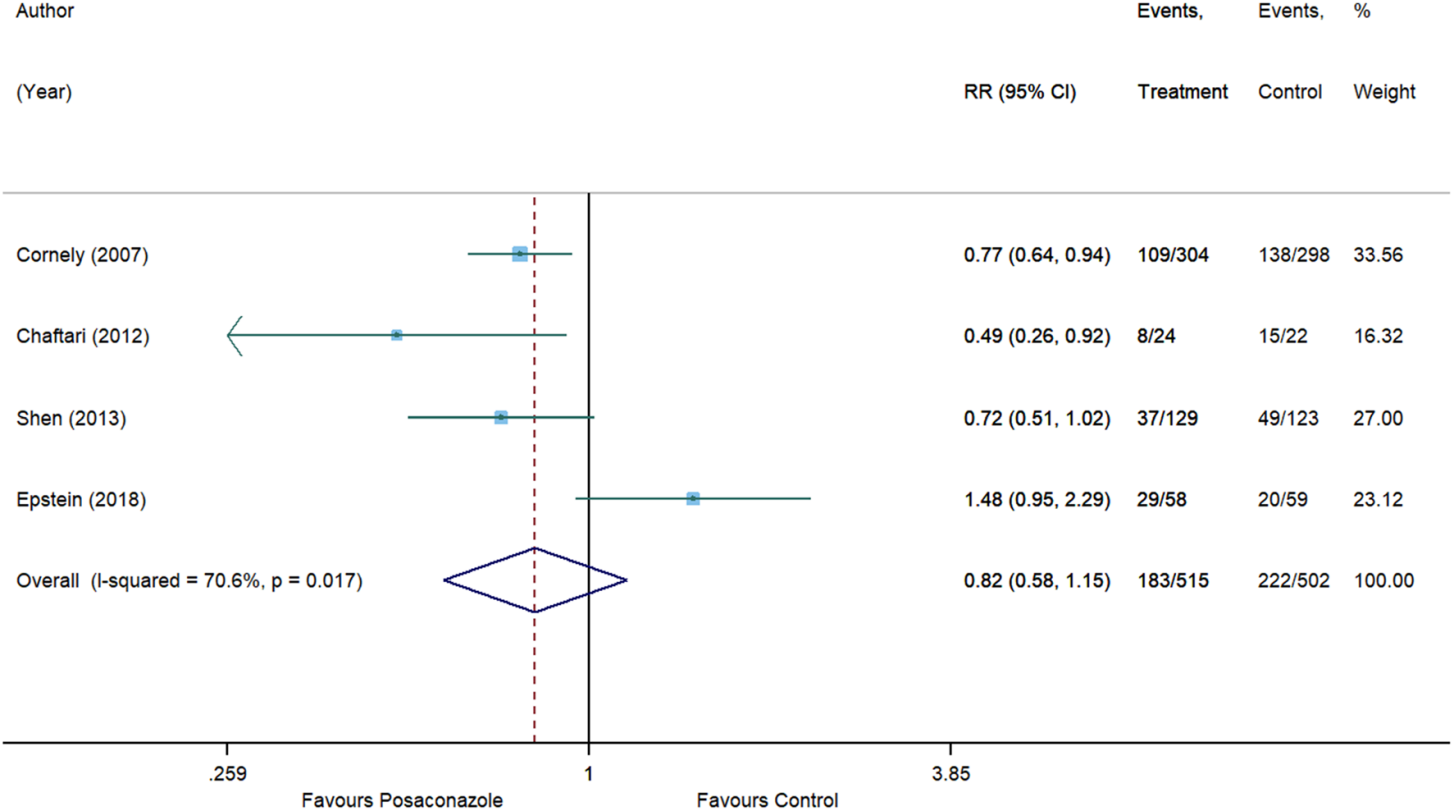
Forest plot and pooled risk estimate for clinical failure comparing posaconazole with other antifungal agents. RR, relative risk; 95% CI, 95% confidence interval.

**Figure 8:**
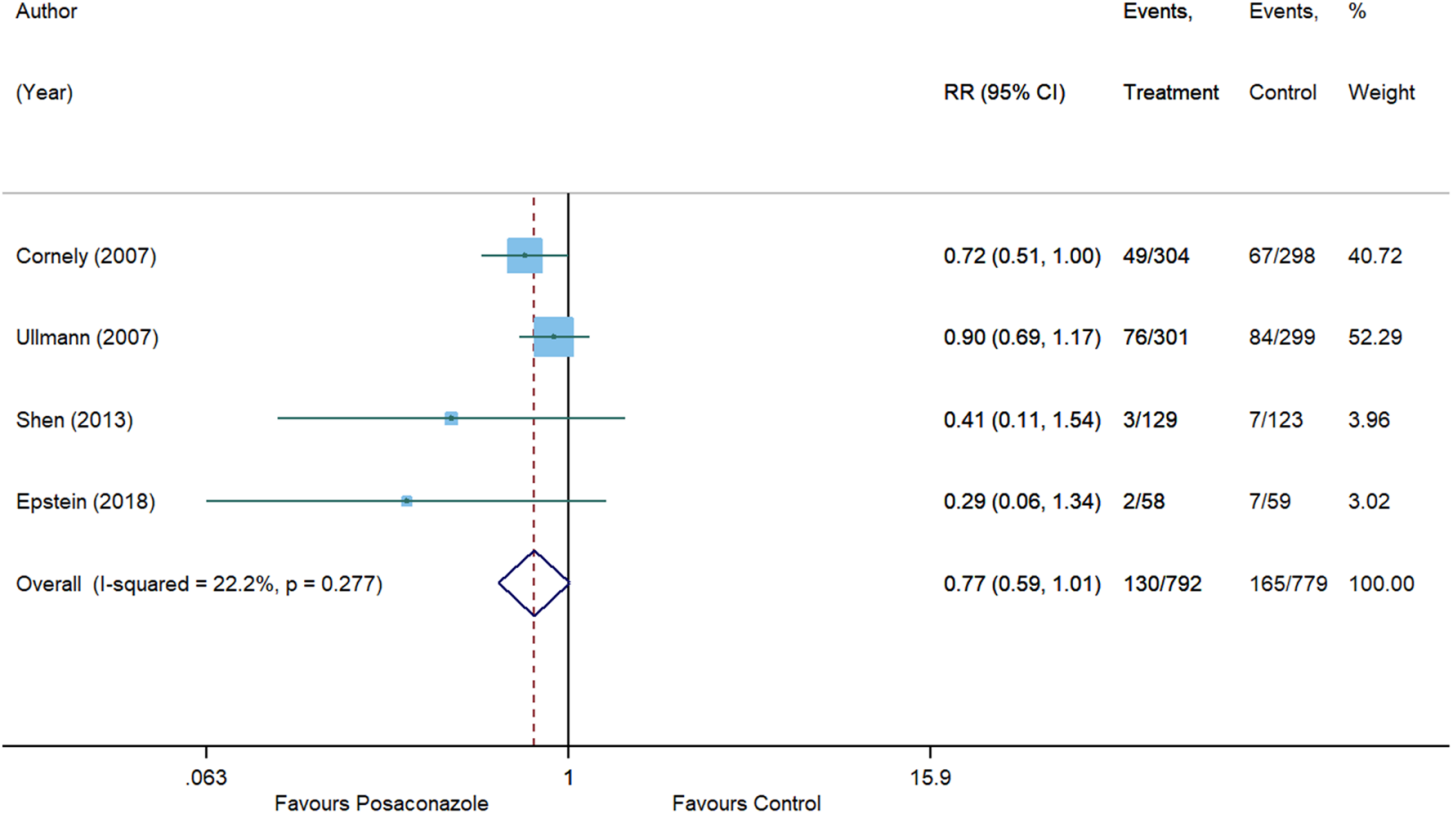
Forest plot and pooled risk estimate for all-cause mortality comparing posaconazole with other antifungal agents. RR, relative risk; 95% CI, 95% confidence interval.

**Figure 9:**
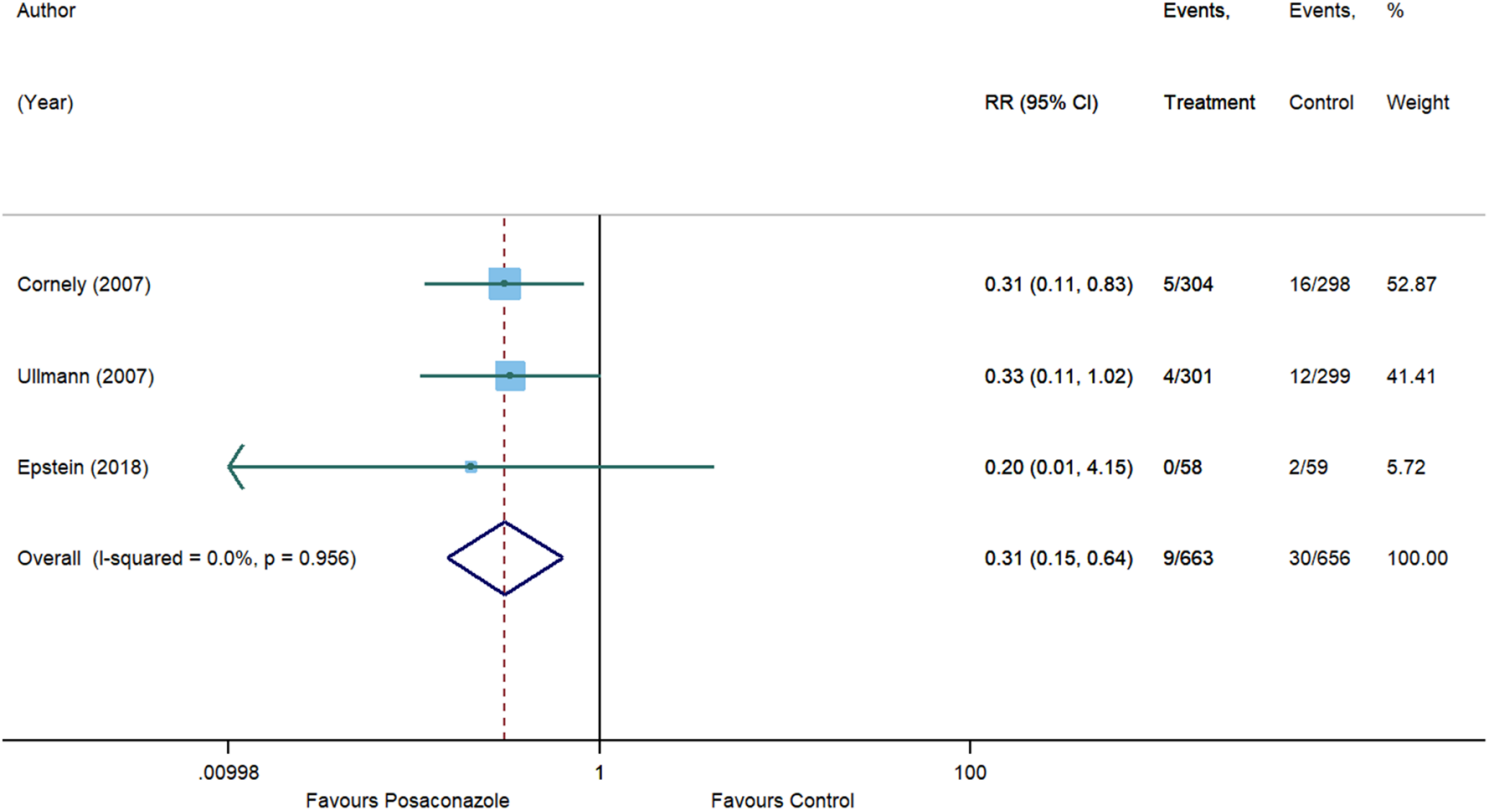
Forest plot and pooled risk estimate for infection-related mortality comparing posaconazole with other antifungal agents. RR, relative risk; 95% CI, 95% confidence interval.

For invasive aspergillosis, posaconazole reduced the risk by 71% (RR, 0.29 [95% CI 0.08 to 1.09], p = 0.066) compared to control. However, statistical significance was not achieved and substantial heterogeneity was detected (I^2^ = 54.7%) (Fig. 5). In comparison with control, no significant associations were also observed for posaconazole in reducing the risk of invasive candidiasis (RR, 1.01 [95% CI 0.36 to 2.84], p = 0.982, I^2^ = 0%) (Fig. 6), clinical failure (RR, 0.82 [95% CI 0.58 to 1.15], p = 0.246, I^2^ = 70.6%) (Fig. 7), and all-cause mortality (RR, 0.77 [95% CI 0.59 to 1.01], p = 0.055, I^2^ = 22.2%) (Fig. 8). Meanwhile, posaconazole demonstrated a significant reduction in infection-related mortality (RR, 0.31 [95% CI 0.15 to 0.64], p = 0.001, I^2^ = 0%) (Fig. 9).

### Safety outcome: treatment-related adverse events

**Figure 10:**
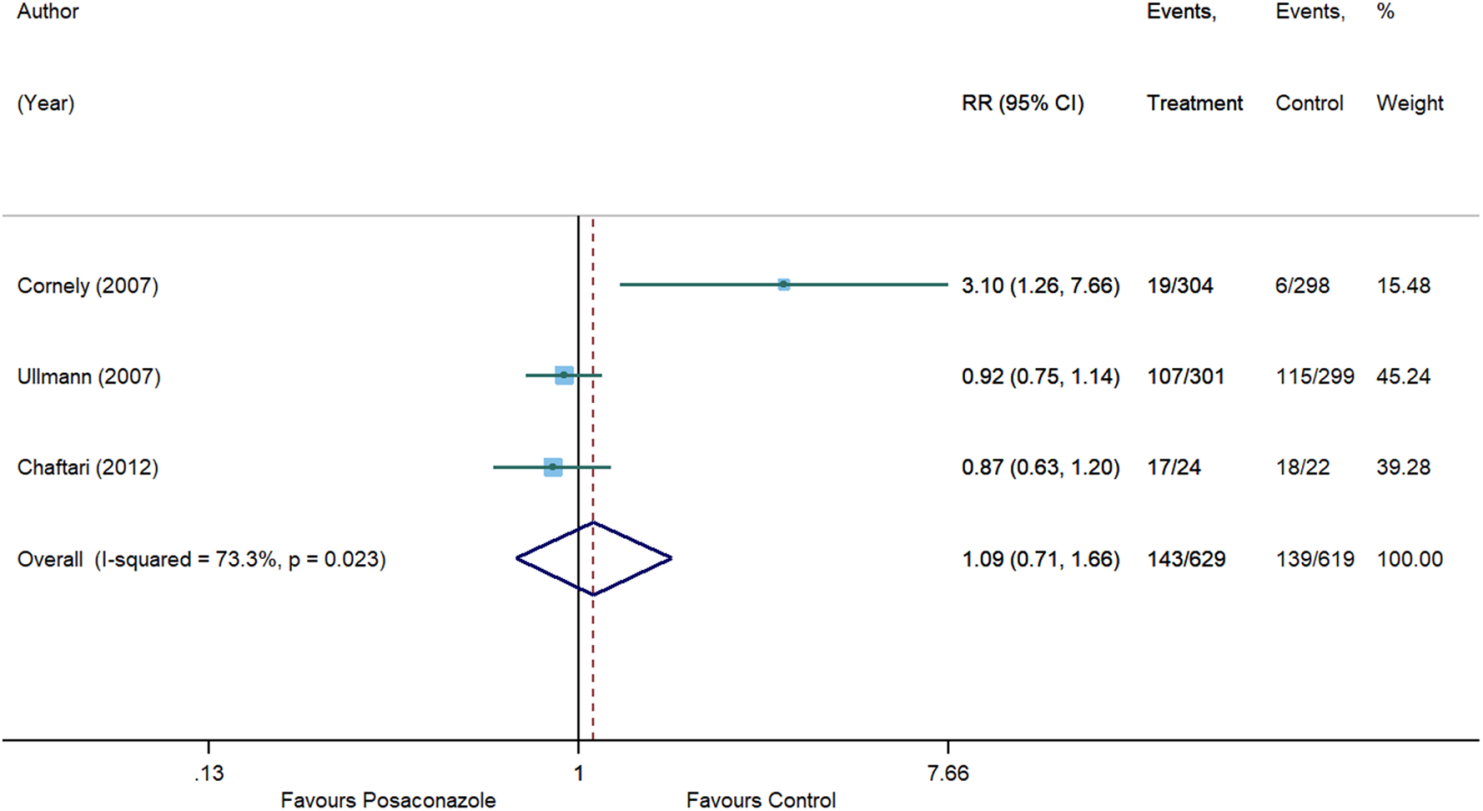
Forest plot and pooled risk estimate for treatment-related adverse events comparing posaconazole with other antifungal agents. RR, relative risk; 95% CI, 95% confidence interval.

Four RCTs^12–15^ provided information on the incidence of treatment-related adverse events. Shen et al^15^ did not report the overall number of patients who experienced adverse events; hence, this study was not included in our meta-analysis. Commonly documented antifungal-related adverse events were gastrointestinal disorders (nausea, vomiting, and diarrhea) and abnormalities in liver function. No significant difference was detected between posaconazole and other antifungal agents (RR, 1.09 [95% CI 0.71 to 1.66], p = 0.703, I^2^ = 73.3%) (Fig. 10).

### GRADE: summary of evidence for posaconazole

Randomized trials are assigned with high rating provided there are no major study limitations. Most of the included trials^12,13,16^ in our primary meta-analysis had some concerns of bias due to the lack of blinding and allocation concealment. However, the quality of evidence remained unaffected as the overall limitations were not serious. No inconsistency was observed among trials included in the primary meta-analysis. In addition to different contributory factors to the risk of neutropenia, the use of interventions at different doses with variable duration of treatment across the studies suggested potential indirectness. The results of TSA indicated that the optimal information size was achieved and the 95% CI excluded the value of no effect.

We thereby concluded that the evidence for posaconazole in preventing IFIs among immunocompromised patients was of high quality. Table 2 shows the GRADE evidence and summary of findings for our primary outcome (refer to Supplementary material, Table 3 for other outcomes).

**Table 2:** GRADE summary of findings for primary outcome.

Certainty assessment							No. of patients		Effect		Certainty	Importance
No. of studies	Study design	Risk of bias	Inconsistency	Indirectness	Imprecision	Other considerations	Posaconazole	Control	Relative (95% CI)	Absolute (95% CI)		
**Posaconazole prophylaxis and the incidence of invasive fungal infections (follow-up: range 5 weeks to 16 weeks)**												
5	Randomized trials	Not serious	Not serious	Serious^a^	Not serious	Strong association	28/816 (3.4%)	67/801 (8.4%)	RR 0.43 (0.28 to 0.66)	48 fewer per 1,000 (from 60 to 28 fewer)	⨁⨁⨁⨁ HIGH	Critical

RR, relative risk; 95% CI, 95% confidence interval.

^a^The contributory factor to the risk of neutropenia differed across trials as study participants received either chemotherapy or hematopoietic stem cell transplantation (HSCT), whereby some HSCT recipients developed graft-versus-host disease and were treated with immunosuppressive agents. In the treatment arm, the dose of posaconazole used was not consistent across all trials. In the control arm, different types of interventions at different doses were used. The duration of treatment phase also varied across studies.

## Discussion

IFIs have long been recognized as a contributory factor to the significant increase in morbidity and mortality among immunocompromised patients, hence antifungal prophylaxis is of great importance in high-risk patients^3^. Posaconazole is a relatively potent triazole with promising antifungal activities against a wide array of fungal species^6–8^. Recent network meta-analyses ranked posaconazole highly for its efficacy in reducing the overall incidence of IFIs^29–32^. Similarly, the results of the present meta-analysis suggested that posaconazole significantly lowered the risk of IFIs as compared to control, with conclusive and high-quality evidence. This study also showed that posaconazole was beneficial in reducing infection-related mortality.

The findings from the subgroup analysis suggested that posaconazole was significantly superior to fluconazole in decreasing the risk of IFIs. Based on two of the studies^12,13^ included in the subgroup analysis, the incidence of invasive aspergillosis was significantly greater among patients who received fluconazole. This may be attributable to the selective antifungal activity of fluconazole against yeast pathogens, limiting its role in preventing invasive mold infections^33^, which translates into lower overall incidence of IFIs in patients on posaconazole prophylaxis. In clinical settings, fluconazole is one of the commonly used antifungal agents due to excellent tolerability and its high bioavailability^34^. However, the widespread use of fluconazole has driven the pathogenic shift to resistant strains of non-*albicans Candida*^35,36^. Moreover, the prophylactic role of fluconazole may be progressively diminishing owing to the rising incidence of invasive mold infections, notably invasive aspergillosis in HSCT recipients^10,37^. In light of the evolving epidemiological trends of IFIs, posaconazole stands out as a suitable antifungal agent for primary prophylaxis.

Other triazoles including itraconazole and voriconazole have also been recommended for the prevention of IFIs^38–41^. However, the use of both agents may be restricted due to poor tolerability and the associated higher incidence of adverse events^33,39,42^. The role of isavuconazole, a relatively new triazole in IFI prophylaxis is less well-studied. To our knowledge, trials are lacking to compare the efficacy and safety of posaconazole with voriconazole and isavuconazole. Echinocandins such as caspofungin, micafungin, and anidulafungin show antifungal properties against *Candida* and *Aspergillus* species with fewer adverse effects or drug interactions^43,44^. In addition to their favourable safety profiles, echinocandins have more predictable pharmacokinetics but they require daily intravenous administration^43,44^. In the published reviews, echinocandins were found to have significantly higher treatment success rates than triazoles for prophylaxis, with micafungin being the most studied agent^45,46^. Nevertheless, none of the included studies compared posaconazole to echinocandins. The present meta-analysis only included one comparative study between posaconazole and micafungin, underlining the need for more clinical trials.

Posaconazole administration may be associated with adverse effects such as nausea, vomiting, diarrhea, headache, and abnormalities in liver function^47^. In terms of safety profile, results from the present meta-analysis suggested that there was no significant difference observed between posaconazole and other antifungal agents. Nevertheless, posaconazole should be used with caution in patients with comorbidities due to possible drug–drug interactions^47^. Healthcare providers should also be wary of the potential development of resistance to posaconazole among fungal species^48^.

Currently, posaconazole is available as an oral suspension, delayed-release tablet, and intravenous formulation^47^. Posaconazole oral suspension was used in all of the trials included in this study. The effectiveness of this conventional formulation may be limited by its unpredictable and highly variable bioavailability; hence, it requires multiple daily dosing and concurrent administration with meals to maximize its systemic exposure^49^. In view of the greater bioavailability offered by the two newer formulations^50–53^, posaconazole delayed-release tablet and intravenous formulation are encouraged to be used in future clinical trials to compare their efficacy and safety with other antifungal agents.

There are several limitations to this systematic review. This review identified only five eligible RCTs and some trials involved small number of participants. Moreover, high-quality trials were lacking as those included in this study were mostly open-label trials. The duration of treatment and follow-up period were not identical across studies, which may have affected the summary effect estimates. In addition, the optimal duration of antifungal prophylaxis remained unknown. Posaconazole prophylaxis in other immunocompromised populations such as solid organ transplant recipients and autologous HSCT recipients were not discussed in the current review due to the absence of RCTs. The prophylactic role of posaconazole in allogenic HSCT recipients without GVHD and patients with hematologic malignancies other than acute myeloid leukemia and myelodysplastic syndrome were also less clear.

## Conclusions

The present meta-analysis supports the use of posaconazole for IFI prophylaxis in patients with hematologic malignancies (particularly acute myeloid leukemia and myelodysplastic syndrome) and allogenic HSCT recipients with conclusive and high-quality evidence. However, additional well-designed trials are required to study the efficacy and safety of posaconazole delayed-release tablet and intravenous formulation in comparison with other antifungal agents. We also emphasize the need of future clinical trials in other patient settings to extensively study the role of posaconazole in the primary prevention of IFIs.

## Supplementary information

Supplementary Information

## Data Availability

All other data is available in the Supplementary Material and any further information is available upon request from the corresponding author.
