# Supplementary material for: Efficacy and safety of posaconazole for the prevention of invasive fungal infections in immunocompromised patients: a systematic review with meta-analysis and trial sequential analysis
Source: Sci Rep. 2020 Sep 3;10:14575. doi: 10.1038/s41598-020-71571-0 (PMC7471265; doi:10.1038/s41598-020-71571-0)
Supplement: Supplementary file 1 — Supplementary Information [file 41598_2020_71571_MOESM1_ESM.docx]

**Supplementary Material**

**Efficacy and safety of posaconazole for the prevention of invasive fungal infections in immunocompromised patients: a systematic review with meta-analysis and trial sequential analysis**

Tse Yee Wong^1^, Yee Shen Loo^1^, Sajesh Kalkandi Veettil^2#^, Pei Se Wong^2^, Gopinath Divya^3^, Siew Mooi Ching^4^ and Rohit Kunnath Menon^5^*

^1^ School of Pharmacy, International Medical University, Kuala Lumpur, Malaysia

^2^ Department of Pharmacy Practice, School of Pharmacy, International Medical University, Kuala Lumpur, Malaysia

^3^ Oral Diagnostic and Surgical Sciences, School of Dentistry, International Medical University, Kuala Lumpur, Malaysia

^4^ Department of Family Medicine, Faculty of Medicine and Health Sciences, Universiti Putra Malaysia, Serdang, Malaysia

^5^ Division of Clinical Dentistry, School of Dentistry, International Medical University, Kuala Lumpur, Malaysia

* Corresponding author

^#^ Co-corresponding author

**Table 1. Search strategy.**

| **No.** | **Search Term** | **Search Results** | | |
| --- | --- | --- | --- | --- |
|  |  | **Embase 1947** | **CENTRAL** | **MEDLINE** |
| 1 | exp Neoplasms/ | 4875367 | 77595 | 3325889 |
| 2 | exp Leukemia/ | 335868 | 4644 | 231006 |
| 3 | exp Lymphoma/ | 324641 | 3173 | 170899 |
| 4 | exp Radiotherapy/ | 598222 | 5856 | 184472 |
| 5 | chemotherapy.mp.  *exp Chemotherapy/ | 668787* | 78184 | 434567 |
| 6 | exp Bone Marrow Transplantation/ | 67691 | 1368 | 44508 |
| 7 | exp Hematopoietic Stem Cell Transplantation/ | 64495 | 1149 | 44651 |
| 8 | exp Transplants/ | 1100068 | 426 | 23421 |
| 9 | exp Graft vs Host Disease/ | 66573 | 656 | 22792 |
| 10 | exp Immunocompromised Host/ | 18616 | 243 | 24637 |
| 11 | exp Myelodysplastic Syndromes/ | 44711 | 684 | 20772 |
| 12 | chemo$.ti,ab. | 985410 | 78069 | 640158 |
| 13 | (radio$ or radia$ or irradiat$).ti,ab. | 2155894 | 92051 | 1501435 |
| 14 | (bone adj marrow adj5 transplant$).ti,ab. | 52963 | 2746 | 37801 |
| 15 | (tumor$ or tumour$).ti,ab. | 2393918 | 66224 | 1651897 |
| 16 | neoplasm$.ti,ab. | 194600 | 5965 | 132404 |
| 17 | cancer$.ti,ab. | 2552326 | 148572 | 1718943 |
| 18 | (leukaemi$ or leukemi$).ti,ab. | 375354 | 13403 | 261299 |
| 19 | malignan$.ti,ab. | 852165 | 25094 | 559567 |
| 20 | carcino$.ti,ab. | 1111948 | 33219 | 779867 |
| 21 | lymphoma$.ti,ab. | 258803 | 9981 | 173913 |
| 22 | exp Invasive Fungal Infections/  ^§^invasive fungal infection.mp. | 18315 | 168^§^ | 6493 |
| 23 | exp Aspergillosis/  *exp Invasive Aspergillosis/ | 4173* | 154 | 16293 |
| 24 | exp Candidiasis, Invasive/  *exp Invasive Candidiasis/ | 2972* | 54 | 1773 |
| 25 | (mycosis or mycotic).ti,ab. | 24771 | 628 | 16481 |
| 26 | posaconazole.mp.  *exp Posaconazole/ | 8271* | 186 | 2638 |
| 27 | exp Randomized Controlled Trial/ | 610124 | 131 | 507823 |
| 28 | exp Clinical Trial/ | 1518209 | 163 | 860755 |
| 29 | randomized controlled trial.pt. | 0 | 495880 | 506996 |
| 30 | random$.tw. | 1553422 | 956392 | 1111699 |
| 31 | blind$.ti,ab. | 434474 | 319311 | 291725 |
| 32 | placebo$.ti,ab. | 314382 | 298344 | 212052 |
| 33 | trial$.ti,ab. | 1477629 | 652484 | 1004417 |
| 34 | or/1-21 | 8514569 | 292484 | 5593610 |
| 35 | or/22-25 | 42367 | 982 | 36785 |
| 36 | or/27-33 | 3276846 | 1236237 | 2161462 |
| 37 | 26 and 34 and 35 and 36 | 350 | 20 | 44 |
| 38 | ^#^limit 37 to humans  *limit 37 to humans and exclude  MEDLINE journals | 44* | 20 | 43^#^ |

**Fig. 1. Risk of bias of included trials.**


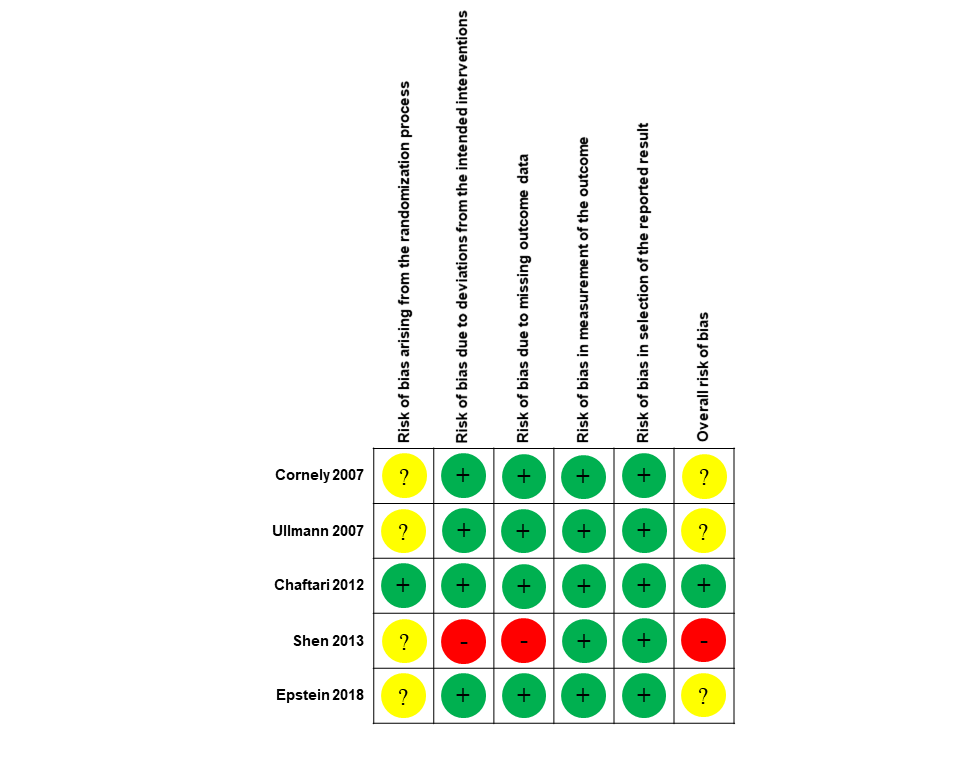


**Fig. 2.** **Sensitivity analysis by using fixed-effects model.** RR, relative risk; 95% CI, 95% confidence interval.


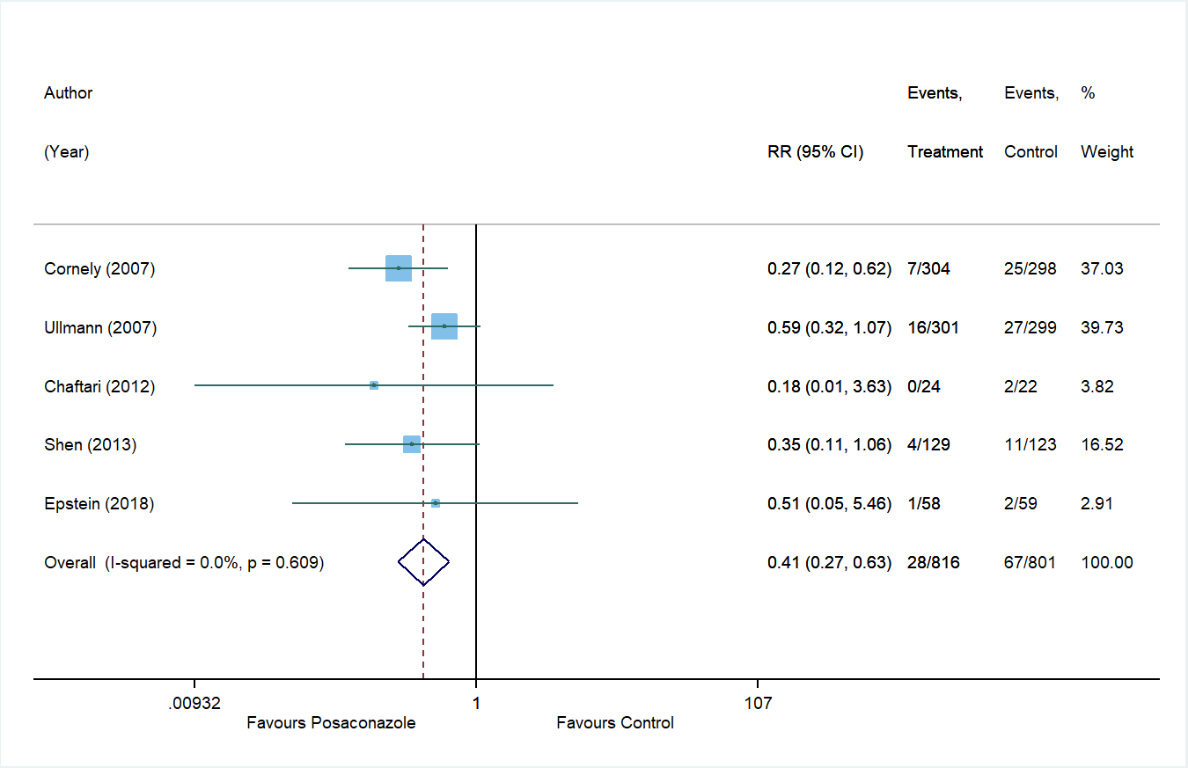


**Fig. 3.** **Sensitivity analysis by excluding trial with high risk of bias.** RR, relative risk; 95% CI, 95% confidence interval.


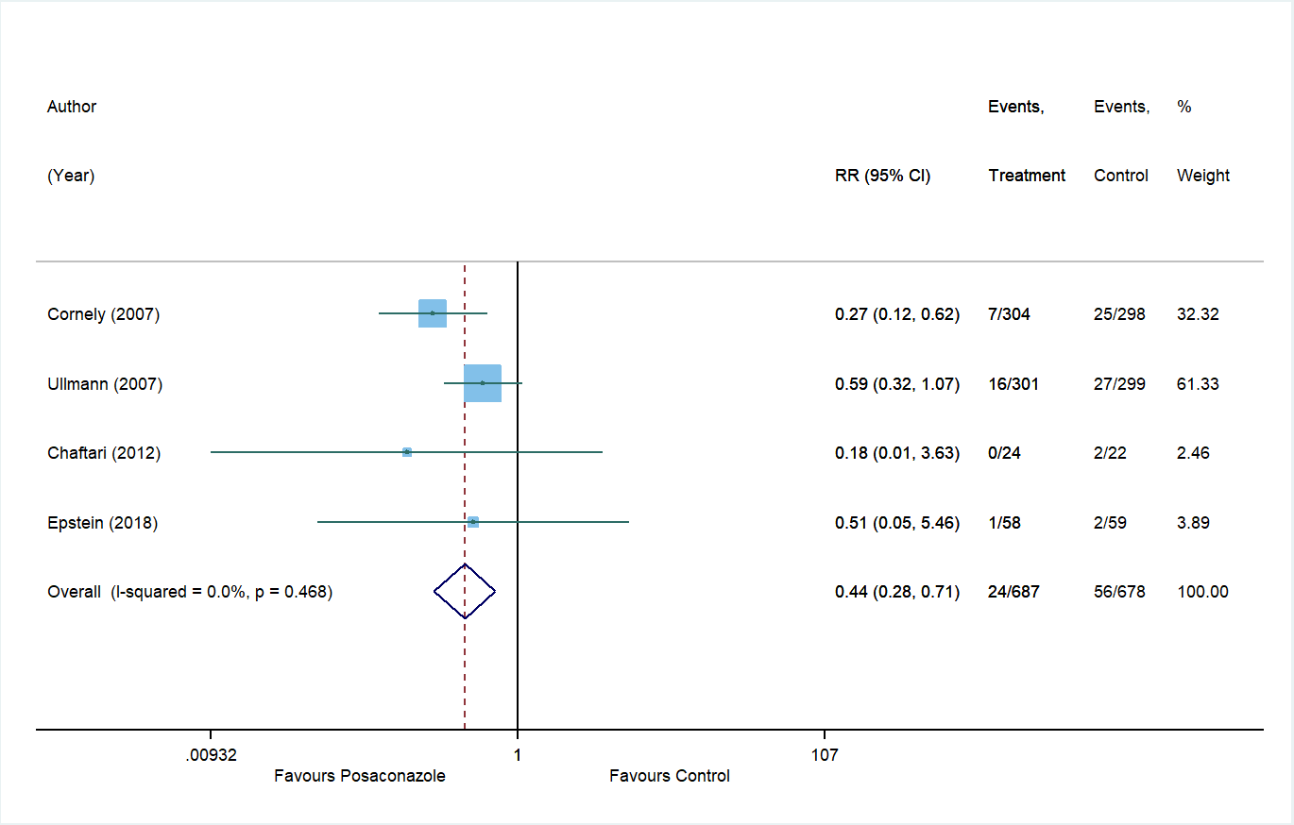


**Fig. 4.** **Funnel plot of studies investigating the effect of posaconazole prophylaxis on the incidence of invasive fungal infections.**

**
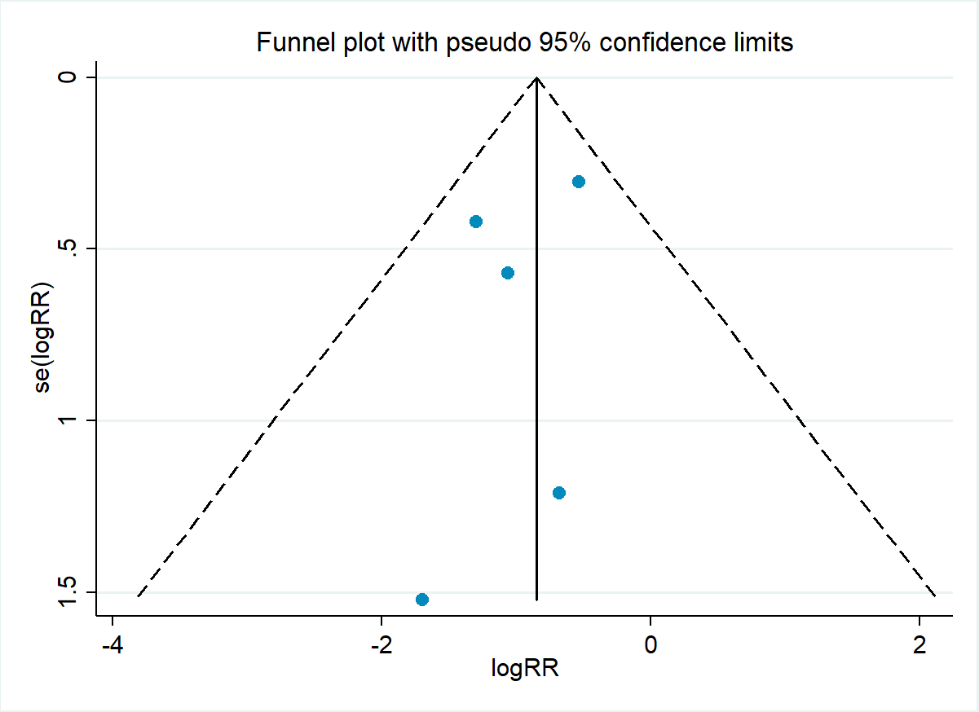
**

**Table 2. Assessment of small-study effects by using Egger’s regression test.**

**Table 3. GRADE summary of findings for secondary outcomes.**

| **Certainty assessment** | | | | | | | **No. of patients** | | **Effect** | | **Certainty** | **Importance** |
| --- | --- | --- | --- | --- | --- | --- | --- | --- | --- | --- | --- | --- |
| **No. of studies** | **Study design** | **Risk of bias** | **Inconsistency** | **Indirectness** | **Imprecision** | **Other considerations** | **Posaconazole** | **Control** | **Relative (95% CI)** | **Absolute (95% CI)** |  |  |
| **Incidence of invasive aspergillosis (follow-up: range 5 weeks to 16 weeks)** | | | | | | | | | | | | |
| 3 | randomized trials | not serious | serious^a^ | serious^b^ | serious^c^ | strong association | 10/663  (1.5%) | 41/656 (6.3%) | **RR 0.29** (0.08 to 1.09) | **44 fewer per 1,000** (from 58 fewer to 6 more) | ⨁⨁◯◯ LOW | CRITICAL |
| **Incidence of invasive candidiasis (follow-up: range 5 weeks to 16 weeks)** | | | | | | | | | | | | |
| 3 | randomized trials | not serious | not serious | serious^b^ | serious^c^ | none | 7/663  (1.1%) | 7/656 (1.1%) | **RR 1.01** (0.36 to 2.84) | **0 fewer per 1,000** (from 7 fewer to 20 more) | ⨁⨁◯◯ LOW | CRITICAL |
| **Clinical failure (follow-up: range 8 weeks to 16 weeks)** | | | | | | | | | | | | |
| 4 | randomized trials | not serious | very serious^a^ | serious^b^ | serious^c^ | none | 183/515 (35.5%) | 222/502 (44.2%) | **RR 0.82** (0.58 to 1.15) | **80 fewer per 1,000** (from 186 fewer to 66 more) | ⨁◯◯◯ VERY LOW | IMPORTANT |

| **Certainty assessment** | | | | | | | **No. of patients** | | **Effect** | | **Certainty** | **Importance** |
| --- | --- | --- | --- | --- | --- | --- | --- | --- | --- | --- | --- | --- |
| **No. of studies** | **Study design** | **Risk of bias** | **Inconsistency** | **Indirectness** | **Imprecision** | **Other considerations** | **Posaconazole** | **Control** | **Relative (95% CI)** | **Absolute (95% CI)** |  |  |
| **All-cause mortality (follow-up: range 12 weeks to 24 weeks)** | | | | | | | | | | | | |
| 4 | randomized trials | not serious | not serious | serious^b^ | serious^c^ | none | 130/792 (16.4%) | 165/779 (21.2%) | **RR 0.77** (0.59 to 1.01) | **49 fewer per 1,000** (from 87 fewer to 2 more) | ⨁⨁◯◯ LOW | CRITICAL |
| **Infection-related mortality (follow-up: range 12 weeks to 24 weeks)** | | | | | | | | | | | | |
| 3 | randomized trials | not serious | not serious | serious^b^ | not serious | strong association | 9/663  (1.4%) | 30/656 (4.6%) | **RR 0.31** (0.15 to 0.64) | **32 fewer per 1,000** (from 39 fewer to 16 fewer) | ⨁⨁⨁⨁ HIGH | CRITICAL |
| **Treatment-related adverse events (follow-up: range 8 weeks to 24 weeks)** | | | | | | | | | | | | |
| 3 | randomized trials | not serious | very serious^a^ | serious^b^ | serious^c^ | none | 143/629 (22.7%) | 139/619 (22.5%) | **RR 1.09** (0.71 to 1.66) | **20 more per 1,000** (from 65 fewer to 148 more) | ⨁◯◯◯ VERY LOW | IMPORTANT |

**Abbreviations:** RR, relative risk; 95% CI, 95% confidence interval

**Explanations**

1. Inconsistency was explained by I^2^ statistic: incidence of invasive aspergillosis (I^2^=54.7%); clinical failure (I^2^=70.6%); treatment-related adverse events (I^2^=73.3%).
2. The contributory factor to the risk of neutropenia differed across trials as study participants received either chemotherapy or hematopoietic stem cell transplantation (HSCT), whereby some HSCT recipients developed graft-versus-host disease and were treated with immunosuppressive agents. In the treatment arm, the dose of posaconazole used was not consistent across all trials. In the control arm, different types of interventions at different doses were used. The duration of treatment and follow-up period also varied across studies.
3. 95% CI included the value of no effect “1”.
